# The *GLCCI1* rs37973 variant and the efficacy of inhaled corticosteroids in the treatment of asthma: A meta‐analysis

**DOI:** 10.1111/crj.13627

**Published:** 2023-05-08

**Authors:** Wen‐Ling Feng, Wen Pu, Jing Li, Yuan Yuan, Ming‐Zhi Yan, Shuang‐Li Yuan, Yu‐Kun Li, Jie‐Ru Wu, Shao‐Quan Xu, Jun Zhao

**Affiliations:** ^1^ Department of Pharmacy The First Affiliated Hospital of Xinjiang Medical University Urumqi Xinjiang China; ^2^ State Key Laboratory of Pathogenesis, Prevention, and Treatment of Central Asian High Incidence Diseases, Clinical Medical Research Institute The First Affiliated Hospital of Xinjiang Medical University, Urumqi Xinjiang China; ^3^ College of Pharmacy Xinjiang Medical University Urumqi Xinjiang China

**Keywords:** asthma, *GLCCI1* (rs37973 G > A), inhaled corticosteroids, meta‐analysis, variant

## Abstract

**Objective:**

This study investigated the relationship between the glucocorticoid‐induced transcript 1 (*GLCCI1*) gene variant and the degree of improvement in lung function with inhaled corticosteroids (ICS).

**Methods:**

We searched the PubMed, Embase, Cochrane Library, CBM, CNKI and Wanfang databases to obtain studies on the *GLCCI1* rs37973 variant and the efficacy of ICS in asthma.

**Results:**

The overall meta‐analysis showed that patients with the GG phenotype (mutant homozygotes) exhibited significantly smaller forced expiratory volume in 1 sec (FEV1) change than patients with the AG phenotype (mutant heterozygous) (MD = −0.08, 95% CI [−0.12, −0.03], *P* = 0.001). Compared with the AA phenotype (wild homozygotes), the GG phenotype (MD = −4.23, 95% CI [−6.09, −2.38], *P* < 0.00001) and AG phenotype (MD = −1.92, 95% CI [−2.35, −1.49], *P* < 0.00001) had significantly smaller FEV1%pred changes. The FEV1 change subgroup analysis showed that the GG phenotype group was smaller than the AA phenotype group at 8 (MD = −0.53, 95% CI [−0.91, −0.14], *P* = 0.007), 12 (MD = −0.16, 95% CI [−0.30, −0.02], *P* = 0.02) and 24 (MD = −0.09, 95% CI [−0.17, −0.01], *P* = 0.02) weeks of treatment; the GG phenotype group was smaller than the AG phenotype group at 12 weeks (MD = −0.08, 95% CI [−0.15, −0.01], *P* = 0.02).

**Conclusion:**

This meta‐analysis suggests that the *GLCCI1* rs37973 variant affects the efficacy of ICS and that the presence of the G allele attenuates the improvement in lung function with ICS.

## Graphical abstract

This meta‐analysis suggests that the *GLCCI1* rs37973 variant had significant influence on ICS therapy in patients with asthma. Compared with the AA and AG group, the GG group had smaller improvement in lung function with ICS. Our meta‐analysis could provide evidence for the optimisation of ICS therapy by *GLCCI1* rs37973 genotyping in clinical practice.

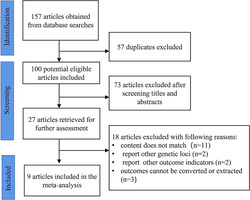

## 1 INTRODUCTION

Asthma is a chronic inflammatory disease of the respiratory system with over 300 million sufferers worldwide, and its incidence and prevalence continue to rise, seriously affecting the quality of life for patients.^1^ Currently, inhaled corticosteroids (ICS) are recognized as the first‐line anti‐inflammatory treatment for asthma.^2^ ICS can alleviate the clinical symptoms of asthma, improve pulmonary function, and suppress airway inflammation.^3^, ^4^ However, there is a significant individual heterogeneity among asthma patients upon ICS treatment.^5^, ^6^, ^7^ Clinical studies have found that 22% of asthma patients do not experience significant improvements in lung function after 12 weeks of ICS treatment.^8^ Additionally, 5% of asthma patients administered with a prescribed dose show a poor response to ICS and require an increased inhaled dose or oral glucocorticoids to control their asthma symptoms.^9^ With the development of pharmacogenomics, genetic differences in individuals are one of the main reasons for the variability in the efficacy of ICS.^10^ Patients with poor response to ICS may have mutations in their glucocorticoid anti‐inflammatory pathway or other pathways that may result in the insensitivity to or the resistance to ICS. Therefore, it is essential to increase their medicine dose or change drugs by using genetic results to predict ICS treatment response.^11^

Glucocorticoid‐induced transcript 1 (*GLCCI1*), an early marker of glucocorticoid‐induced inflammatory cell apoptosis, is associated with asthma susceptibility and glucocorticoid efficacy and is an important gene in the process of glucocorticoid‐promoted inflammatory cell apoptosis.^12^, ^13^ When *GLCCI1* is mutated, it may reduce the efficacy of the ICS response.^14^ A study has shown that the rs37973 variant was significantly associated with improved lung function in patients treated with ICS for 4–8 weeks, and patients carrying the AA genotype showed better improvement in lung function with ICS.^15^ In the Chinese Han population, patients who were homozygotes for the G allele at rs37973 showed significantly less improvement in lung function than both heterozygotes and homozygotes for the A allele after 12 weeks of ICS treatment.^16^ Interestingly, the *GLCCI1* rs37973 variant was not associated with the asthma risk in the Saudi Arabian population.^17^ In addition, another study found that *GLCCI1* rs37973 did not influence the treatment response to ICS in white asthma patients.^18^ Overall, the relevance of the *GLCCI1* rs37973 variant to the efficacy of ICS remains controversial.

Therefore, this study conducted a meta‐analysis of the correlation between the *GLCCI1* rs37973 variant and the efficacy of ICS in the treatment of asthma, providing a theoretical basis for the clinical application of ICS in drug therapy for asthma patients.

## 2 METHODS

### 2.1 Search strategy

**FIGURE 1:**
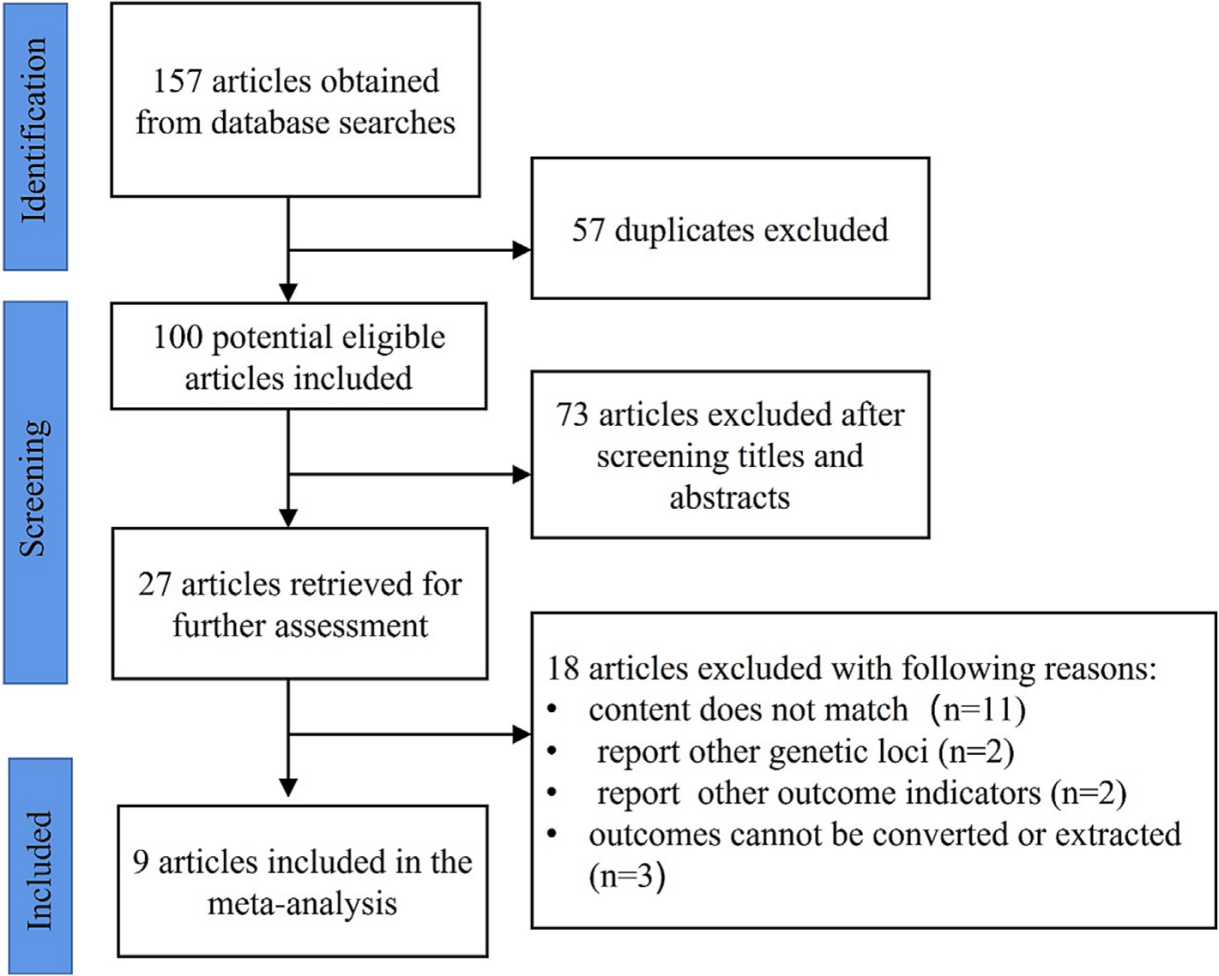
PubMed search strategy.

A comprehensive literature search was carried out in PubMed, Embase, Web of Science, Cochrane Library, and the Chinese National Knowledge Infrastructure databases for all related publications up to February 28, 2022, using the following keyword terms: “asthma”, “inhaled corticosteroids”, “ICS”, “*GLCCI1*” and “Glucocorticoid‐induced transcript 1”, and the related Chinese characters were used as the criteria for searching. No language or publication year was limited. The specific search strategy using PubMed as an example is shown in Figure 1.

### 2.2 Inclusion and exclusion criteria

The inclusion criteria are as follows: (1) observational studies (cohort, cross‐sectional or case–control); (2) evaluated the association between *GLCCI1* gene variants and ICS therapeutic effectiveness in asthma; (3) provided the distribution of genotypes; (4) used the change in the forced expiratory volume in 1 second (FEV1) or FEV1 percentage of predicted value (FEV1%pred) as the endpoints and (4) the number of genotypes conformed to Hardy–Weinberg equilibrium.

Meanwhile, the exclusion criteria are: (1) studies conducted on unrelated diseases such as seasonal allergic rhinitis, chronic obstructive pulmonary disease, nephrotic syndrome and rheumatoid arthritis; (2) studies with inappropriate article types (animal or cell line research, reviews, letters, case reports or abstracts) and (3) research without full text, incomplete information or inability to conduct data extraction.

### 2.3 Data extraction and quality assessment

Two independent authors (J.R.W. and S.Q.X.) carried out the data extraction. A discussion was applied when there was a discrepancy. The following information was extracted: the first author's name, publication year, treatment method, follow‐up time, genotype distribution, number of people between groups, FEV1 and FEV1%pred. A third reviewer (M.Z.Y.) was consulted for the final decision in case of any disagreement on the eligibility between the first two reviewers. We assessed the quality of each included study using the Newcastle–Ottawa Scale (NOS).^19^ The NOS values range from 0 to 9. Studies with a score of 6 are considered to be of high quality.^20^

### 2.4 Statistical analysis

The meta‐analysis was conducted with the Cochrane Collaboration Review Manager 5.4 software (http://tech.cochrane.org/revman). *P <* 0.05 was considered to indicate a statistically significant difference. FEV1 and FEV1%pred were evaluated using the mean difference (MD) with 95% confidence intervals (CIs). The heterogeneity of the included studies was tested using the *χ* ^*2*^ test and *I* ^2^%, and if there was significant heterogeneity between studies (*I* ^2^% > 50%, *P* heterogeneity <0.05), a random‐effects model was used. Otherwise, a fixed model was used. To better apply the results of this study to clinical practice, for all outcomes, treatment follow‐up times were analysed in subgroups.

### 2.5 Sensitivity analysis and publication bias assessment

A sensitivity analysis was performed to assess the influence of each study on the overall result by stepwise eliminating every study individually. Funnel plot analysis and Egger's test were performed to check for publication bias. STATA 14 software was used to finish the statistical manipulations, and *P* < 0.05 was deemed to be statistically significant.

## 3 RESULTS

### 3.1 Characteristics of enrolled studies

After an exhaustive search, a total of 157 publications were identified from the above databases. After the collation through statistics, we removed 57 duplicate articles. After screening titles and abstracts, we identified 27 potentially eligible publications. By reading the full‐text articles, 18 publications were excluded, mainly because *GLCCI1* gene rs37973 locus outcomes were not reported or could not be converted. Therefore, a total of nine publications were included in the meta‐analyses.

**FIGURE 2:**
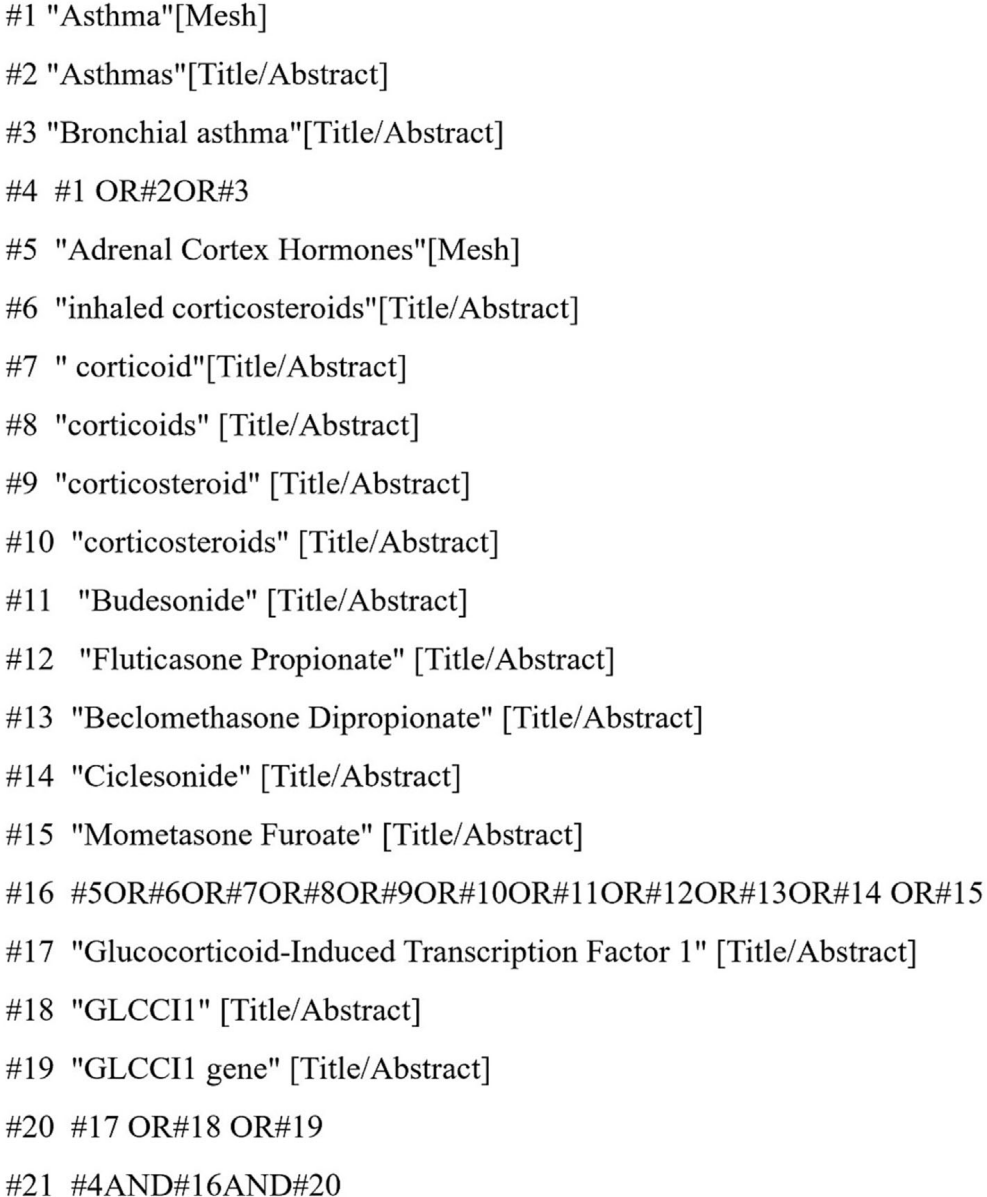
Preferred Reporting Items for Systematic Reviews and Meta‐Analyses (PRISMA) flowchart—selection of studies for meta‐analysis.

Among the nine publications, six reported results for rs37973 genotypes and the amount of FEV1 change, but one of the publications reported data from two different follow‐up times, and we defined the above data from different times as two studies. Seven publications reported results for rs37973 genotypes and in relation to FEV1%pred change. In the above publications, six articles pertained to Asian subjects, two articles pertained to Caucasian subjects, and only one article was Tunisian.^15^, ^16^, ^18^, ^21^, ^22^, ^23^, ^24^, ^25^, ^26^ The details of each publication included in this meta‐analysis are summarized in Table 1 and Figure 2.

**TABLE 1:** Basic characteristics of included studies.

Author	Year	Ethnicity	Sample size	Follow‐up time (week)	Indicators	Genotype			NOS Scores
						AA	AG	GG	
Mariem Salhi^23^	2019	Tunisian	75	12	FEV1; FEV1%pred	27	30	18	7
Louise Hosking^18^	2014	Caucasian	1916	8	FEV1%pred	595	957	364	7
Tantisira KG^15^	2011	Caucasian	935	4–8	FEV1%pred	442	354	139	9
Xu^22^	2017	Asian	418	24	FEV1; FEV1%pred	104	213	101	9
HU^16^	2016	Asian	30	8/12	FEV1	13	13	4	7
Li Jing^24^	2021	Asian	152	12	FEV1	42	72	38	9
Li^25^	2021	Asian	90	8	FEV1; FEV1%pred	26	46	18	9
Qiu^21^	2016	Asian	153	12	FEV1; FEV1%pred	36	91	26	9
Zhu^26^	2021	Asian	60	24	FEV1%pred	19	27	14	9

Abbreviations: FEV1, forced expiratory volume in 1 sec; %pred, percentage of predicted value; NOS, Newcastle‐Ottawa Scale.

### 3.2 Meta‐analysis results

#### 3.2.1 *GLCCI1* (rs37973 G > A) genotypes and FEV1 change

**FIGURE 3:**
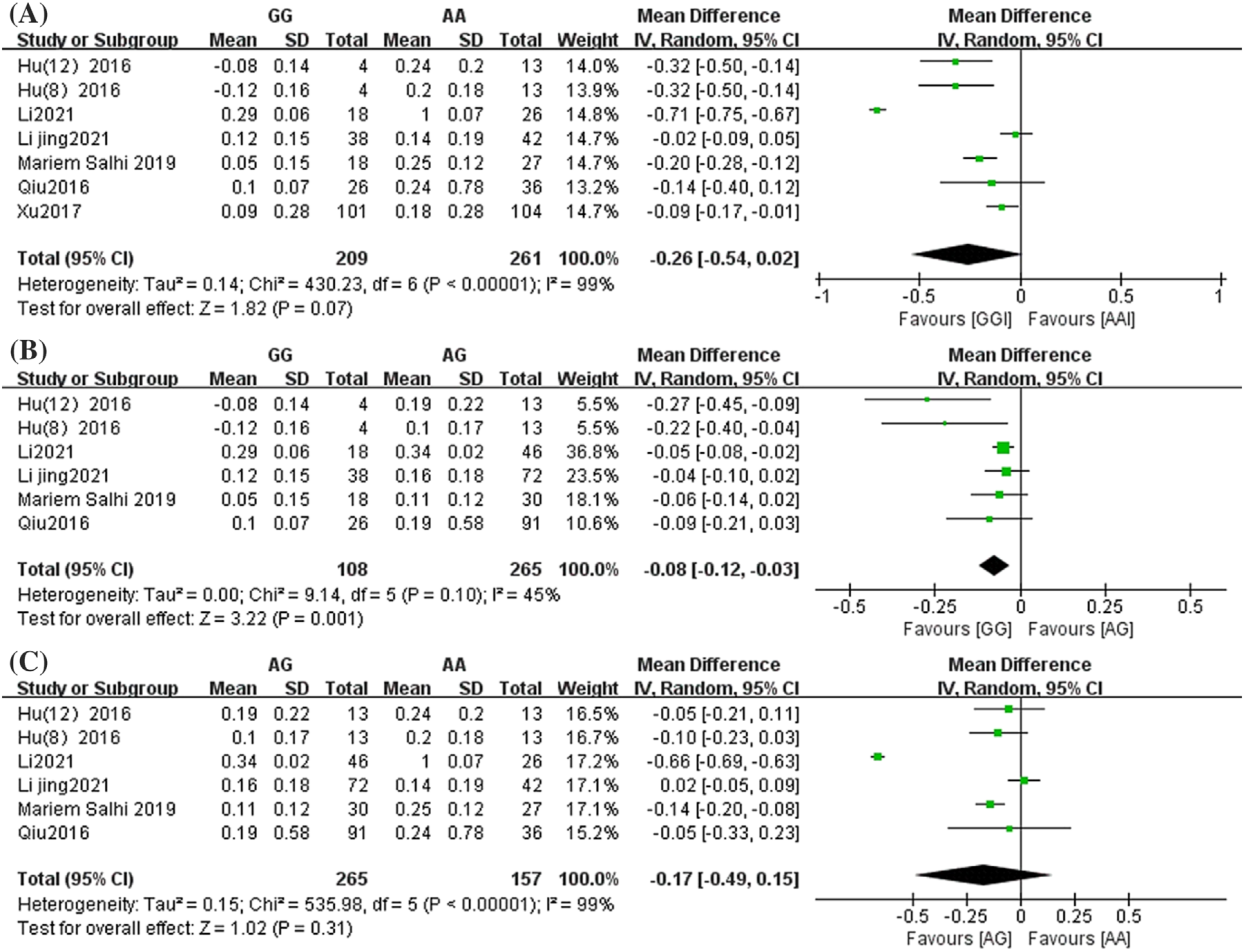
Forest plot of the association between glucocorticoid‐induced transcript 1 (*GLCCI1*) rs37973 genotypes and forced expiratory volume in 1 sec (FEV1) change. (A) GG vs. AA; (B) GG vs. AG; (C) AG vs. AA. SD, standard deviation; IV, inverse variance; CI, confidence interval.

Forest plots comparing the amount of FEV1 change difference between genotypes of rs37973 are shown in Figure 3. There were seven studies involving the amount of change in FEV1 before and after treatment in patients in the GG and AA groups.^16^, ^21^, ^22^, ^23^, ^24^, ^25^, ^26^ They included 209 patients in the GG group and 261 patients in the AA group. The overall analysis results showed that there were no significant differences in the FEV1 change between the GG group and AA group (MD = −0.26, 95% CI [−0.54, 0.02], *P* = 0.07) (Figure 3A). In addition, there were six studies involving the amount of change in FEV1 before and after treatment in patients in the GG, AG and AA groups, with 108 patients in the GG group, 265 patients in the AG group and 157 patients in the AA group.^16^, ^21^, ^23^, ^24^, ^25^ The overall analysis results showed that the change in FEV1 in patients in the GG group was significantly smaller than that in the AG group (MD = −0.08, 95% CI [−0.12, −0.03], *P* = 0.001) (Figure 3B). However, there was no difference in FEV1 change between the AG group and AA group (MD = −0.17, 95% CI [−0.49, 0.15], *P* = 0.31) (Figure 3C).

#### 3.2.2 *GLCCI1* (rs37973 G > A) genotypes and the change in FEV1%pred

**FIGURE 4:**
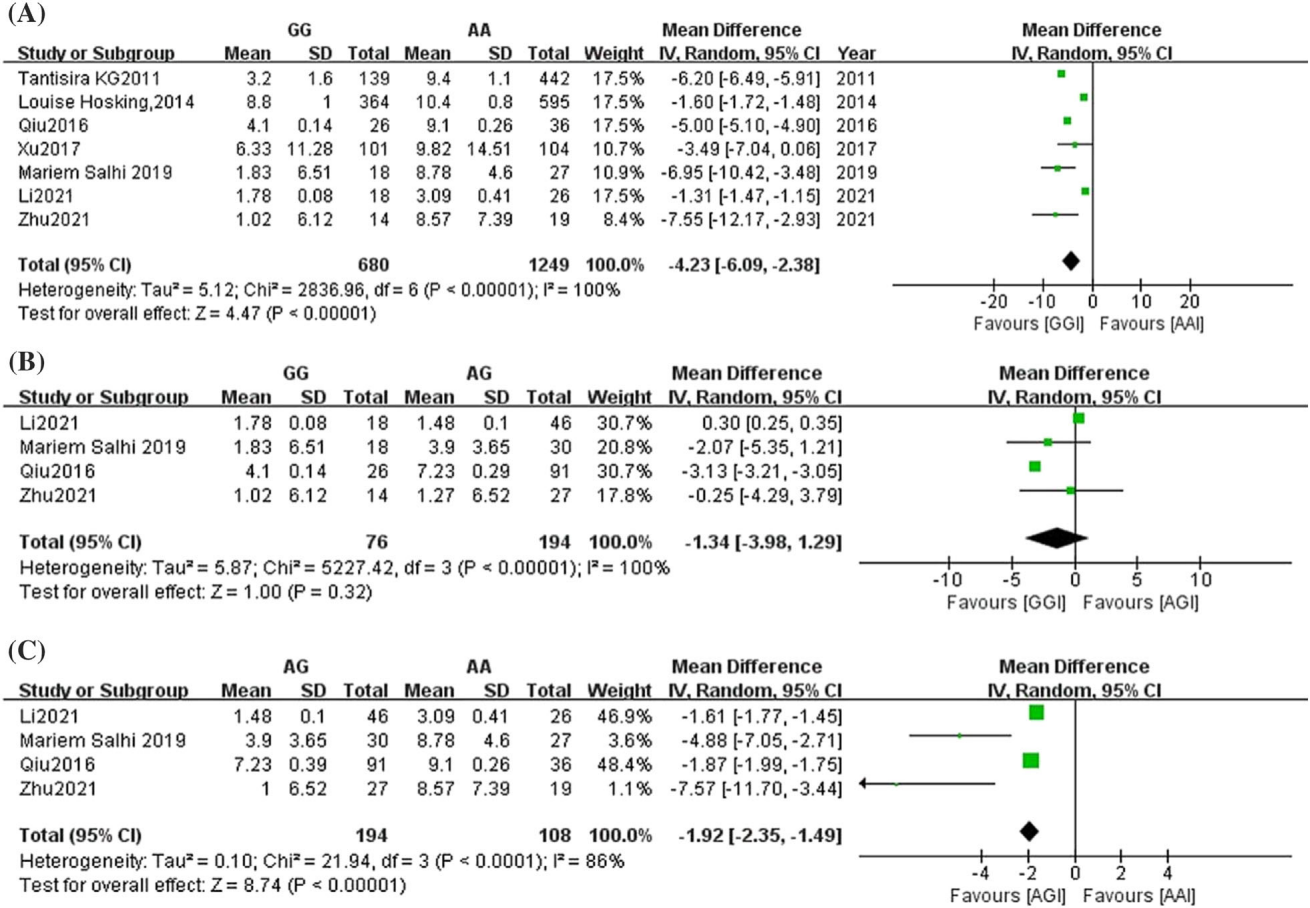
Forest plot of the association between glucocorticoid‐induced transcript 1 (*GLCCI1*) rs37973 genotypes and forced expiratory volume in 1 sec percentage of predicted value (FEV1%pred) change. (A) GG vs. AA; (B) GG vs. AG; (C) AG vs. AA. SD, standard deviation; IV, inverse variance; CI, confidence interval.

Forest plots comparing the FEV1%pred change difference between genotypes of rs37973 are shown in Figure 4. A total of seven studies involved the amount of change in FEV1%pred before and after treatment, with 680 patients in the GG group and 1249 patients in the AA group.^15^, ^18^, ^21^, ^22^, ^24^, ^25^, ^26^ The overall analysis results showed that the change in FEV1%pred in patients in the GG group was significantly smaller than that in patients in the AA group (MD = −4.23, 95% CI [−6.09, −2.38], *P* < 0.00001) (Figure 4A). Four studies addressed the amount of change in FEV1%pred before and after treatment, with 76 patients in the GG group, 194 in the AG group and 108 in the AA group. The results showed that there was no difference in the amount of change in FEV1%pred in asthma patients in the GG group compared with the AG group (MD = −1.34, 95% CI [−3.98, 1.29], *P* = 0.32) (Figure 4B). However, the amount of change in FEV1%pred was significantly lower in the AG group than in the AA group. (MD = −1.92, 95% CI [−2.35, −1.49], *P* < 0.00001) (Figure 4C).

Heterogeneity across studies was observed for both the analysis of FEV1 and FEV1%pred (*I* ^*2*^ > 50%), suggesting a need for subanalyses to address sources of study heterogeneity. Subgroup analyses were conducted based on the differences in the follow‐up time after the treatment with ICS.

#### 3.2.3 Subgroup analysis of the association between *GLCCI1* (rs37973 G > a) genotypes and the FEV1 change

**FIGURE 5:**
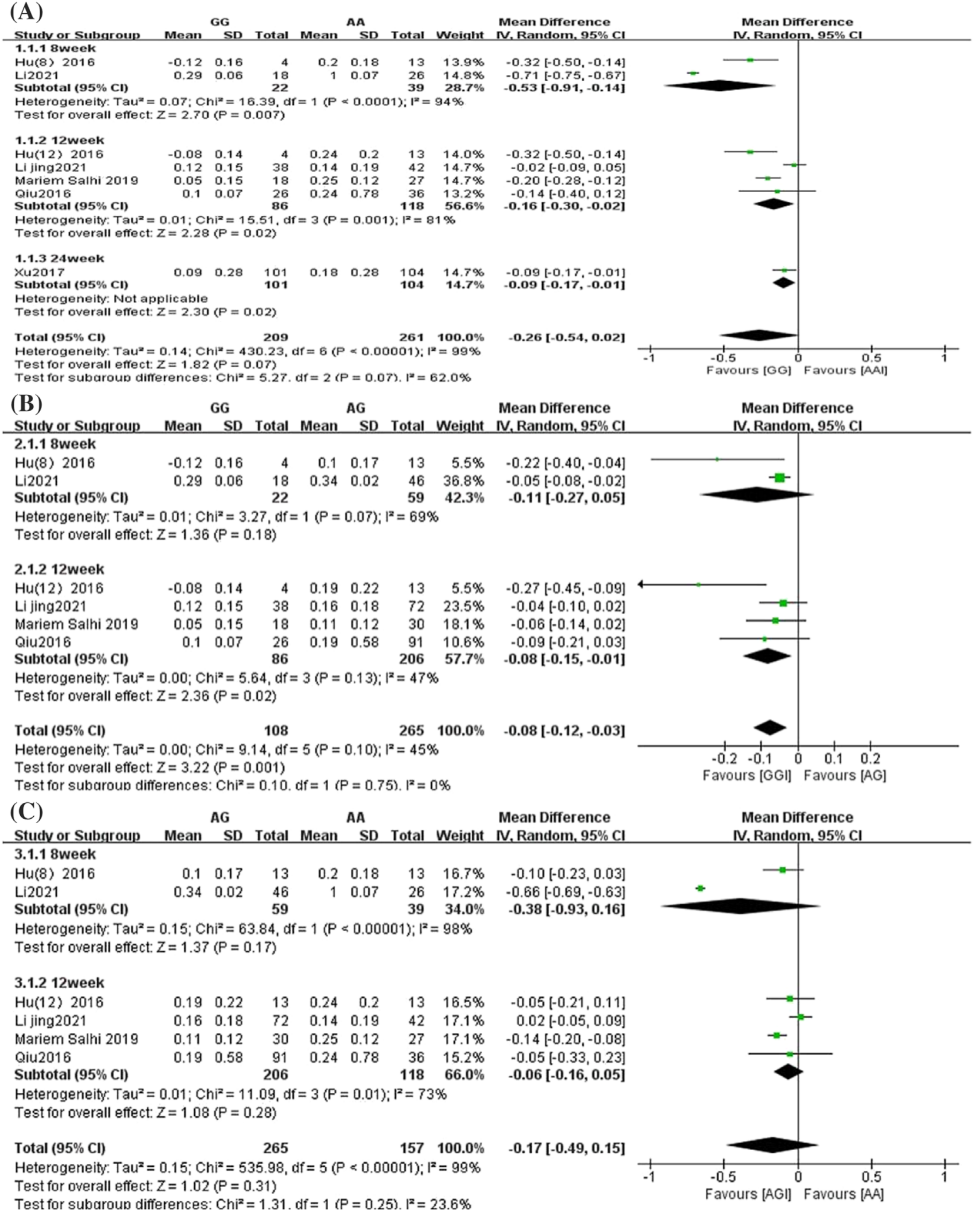
Subgroup analysis of the association between glucocorticoid‐induced transcript 1 (*GLCCI1*) rs37973 genotypes and forced expiratory volume in 1 sec (FEV1) change. (A) GG vs. AA; (B) GG vs. AG; (C) AG vs. AA. SD, standard deviation; IV, inverse variance; CI, confidence interval.

Subgroup analysis forest plots comparing the amount of the FEV1 change difference between genotypes of rs37973 are shown in Figure 5. In the GG and AA group, subgroup analysis was performed according to the duration of treatment with ICS, divided into three subgroups of 8, 12, and 24 weeks of treatment. The results showed that the amount of FEV1 change in the GG group was significantly lower than that in the AA group at 8 (MD = −0.53, 95% CI [−0.91, −0.14], *P* = 0.007), 12 (MD = −0.16, 95% CI [−0.30, −0.02], *P* = 0.02) and 24 (MD = −0.09, 95% CI [−0.17, −0.01], *P* = 0.02) weeks of treatment (Figure 5A). In the GG, AG and AA groups, subgroup analyses were performed according to the duration of treatment with ICS, divided into 8 and 12 weeks of treatment. The results showed that at 8 weeks, there was no difference in the FEV1 change (MD = −0.11, 95% CI [−0.27, 0.05], *P* = 0.18), but at 12 weeks, the amount of FEV1 change was less in the GG group than in the AG group (MD = −0.08, 95% CI [−0.15, −0.01], *P* = 0.02) (Figure 5B). However, at 8 (MD = −0.38, 95% CI [−0.93, 0.16], *P* = 0.17) and 12 weeks of treatment (MD = −0.06, 95% CI [−0.16, 0.05], *P* = 0.28), there was no difference in the FEV1 change between the AG group and AA group (Figure 5C).

#### 3.2.4 Subgroup analysis of the association between *GLCCI1* (rs37973 G > A) genotypes and the FEV1%pred change

**FIGURE 6:**
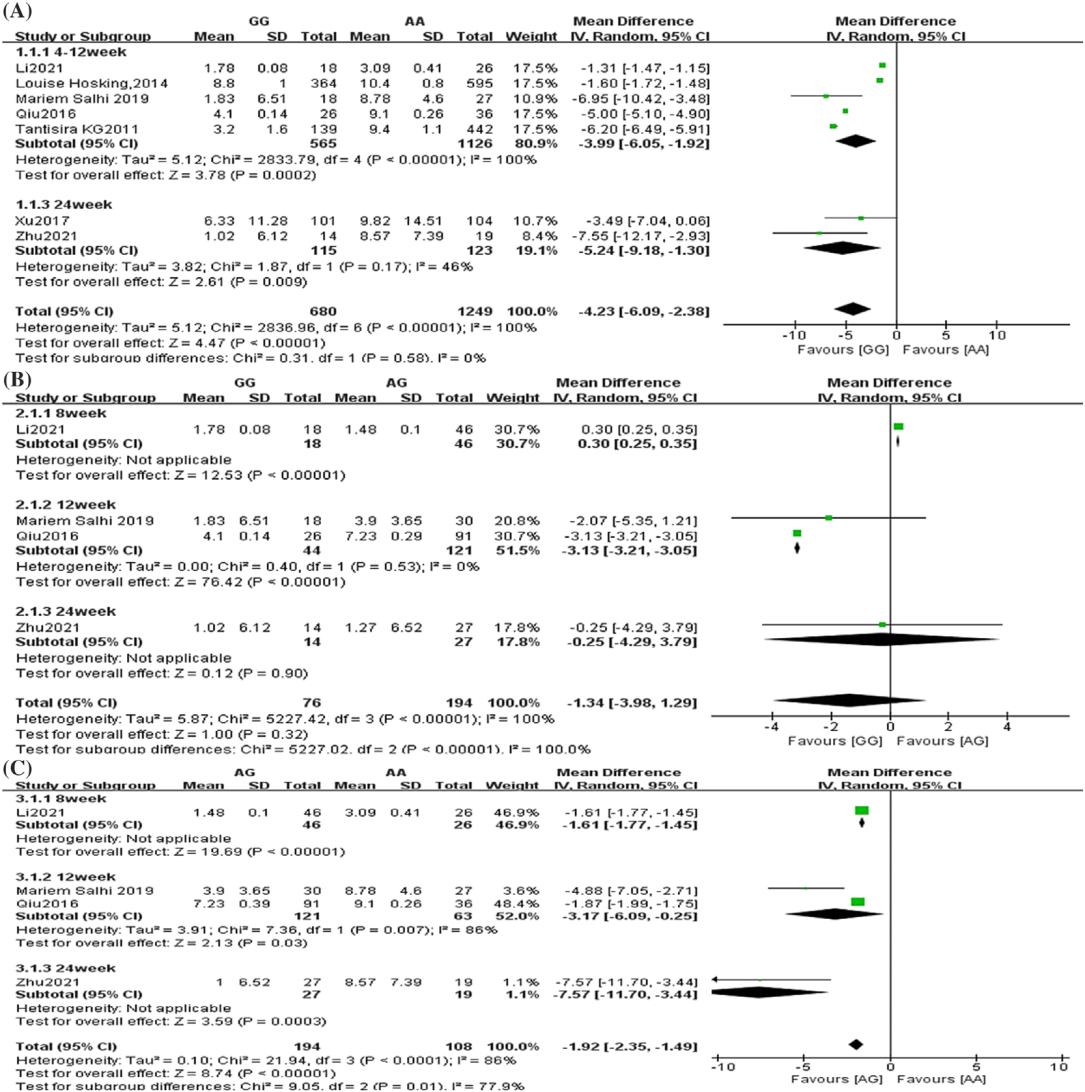
Subgroup analysis of the association between glucocorticoid‐induced transcript 1 (*GLCCI1*) rs37973 genotypes and forced expiratory volume in 1 sec percentage of predicted value (FEV1%pred) change. (A) GG vs. AA; (B) GG vs. AG; (C) AG vs. AA. SD, standard deviation; IV, inverse variance; CI, confidence interval.

Subgroup analysis forest plots comparing the amount of FEV1%pred change difference between genotypes of rs37973 are shown in Figure 6. In the GG and AA groups, subgroup analysis was performed according to the duration of treatment with ICS, divided into two subgroups of 4–12 and 24 weeks of treatment. The results showed that the amount of FEV1%pred change in the GG group was significantly lower than that in the AA group at 4–12 (MD = −3.99, 95% CI [−6.05, −1.92], *P* = 0.0002) and 24 weeks (MD = −5.24, 95% CI [−9.18, −1.30], *P* = 0.009) of treatment (Figure 6A). In the GG and AG groups, the FEV1%pred change of GG was higher than in the AG group at 8 weeks (MD = 0.30, 95% CI [0.25, 0.35], *P* < 0.00001), but at 12 weeks, it was lower in the GG than in the AG group (MD = −3.13, 95% CI [−3.21, −3.05], *P* < 0.00001), and there was no difference at 24 weeks (MD = −0.25, 95% CI [−4.29, 3.79], *P* = 0.90) (Figure 6B). In the AG and AA groups, the change was significantly lower in the AG than the AA group at 8 (MD = −1.61, 95% CI [−1.77, −1.45], *P* < 0.00001), 12 (MD = −3.17, 95% CI [−6.09, −0.25], *P* = 0.03) and 24 weeks (MD = −7.57, 95% CI [−11.70, −3.44], *P* = 0.0003) of treatment (Figure 6C).

### 3.3 Sensitivity analysis and publication bias

**FIGURE 7:**
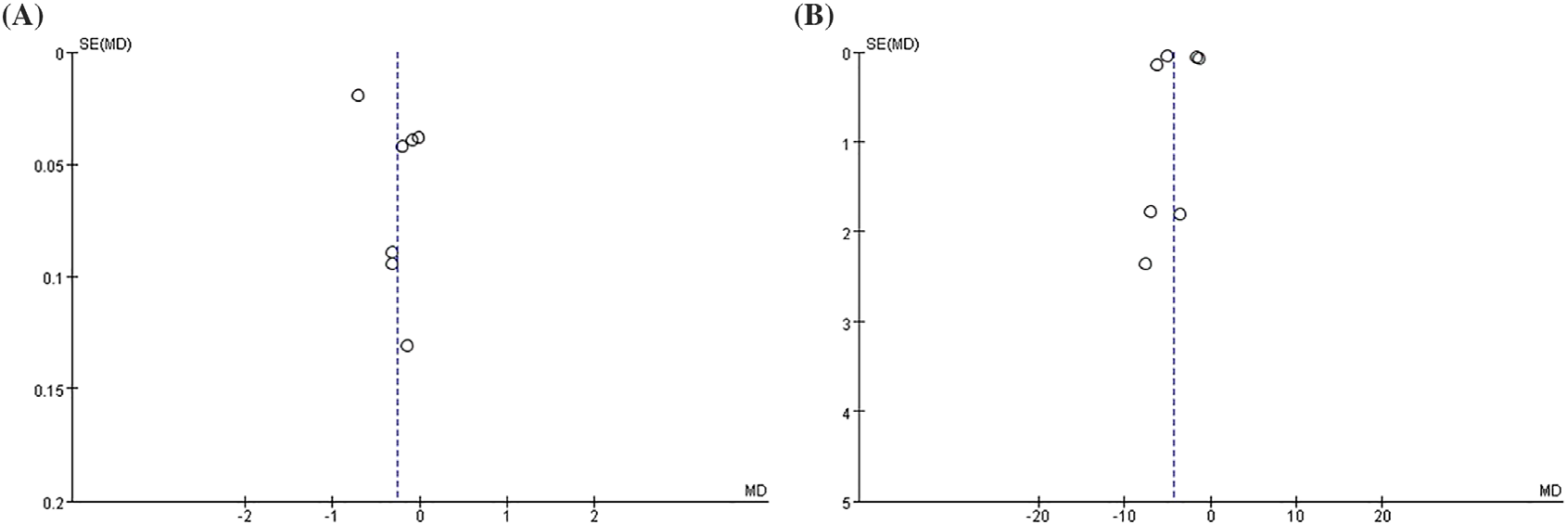
Funnel plot of the glucocorticoid‐induced transcript 1 (*GLCCI1*) rs37973 genotypes and the efficacy of inhaled corticosteroids (ICS). (A) Funnel plot of the rs37973 genotypes and forced expiratory volume in 1 sec (FEV1) (GG vs. AA); (B) funnel plot of the rs37973 genotypes and FEV1 percentage of predicted value (FEV1%pred) (GG vs. AA).

The results suggested that there were no independent studies that significantly influenced the pooled MDs. However, the funnel plot was not perfectly symmetrical. Therefore, there is a potential bias in this systematic evaluation. By Egger's test, in all comparisons in the outcome, the FEV1 and FEV1%pred showed no evidence of publication bias among studies, as shown in Table 2 and Figure 7.

**TABLE 2:** *P* values of the Egger test for publication bias assessment.

Indicator	Comparisons	Egger's test (*P*)
FEV1	GG vs AA	0.170
FEV1	GG vs AG	0.057
FEV1	AG vs AA	0.088
FEV1%pred	GG vs AA	0.961
FEV1%pred	GG vs AG	0.743
FEV1%pred	AG vs AA	0.263

Abbreviations: FEV1, forced expiratory volume in 1 sec; %pred, percentage of predicted value.

## 4 DISCUSSION

Asthma is a complex disease triggered by the interaction between genes and the environment.^27^, ^28^ *GLCCI1* has been identified as a functional variant that plays an important role in the development of asthma as well as in drug efficacy.^15^ In an asthma model, Cheng et al. observed wild‐type mice with more significant relief of airway hyperresponsiveness and inflammation than *GLCCI1*‐deficient mice after administration of glucocorticoids (GCs),^29^ suggesting that *GLCCI1* deficiency may contribute to the inefficiency of GCs in asthma conditions. Furthermore, Cheng et al. also found that the change in *GLCCI1* mRNA expression was significantly positively correlated with the amount of FEV1 change in asthmatic patients treated with ICS in a Chinese Han adult population.^16^ The rs37973 variant is located in the promoter of *GLCCI1*. Tantisira et al., Hu et al. and Xu et al. found that the rs37973 locus variant was associated with reduced ICS response in asthmatic Caucasian, Tunisian and Asian adults.^15^, ^16^, ^22^ Interestingly, in two multicentre, large‐sample studies, the rs37973 locus variant was not found to be associated with the change in the FEV1 after ICS treatment.^18^, ^30^ Additionally, Rijavec et al. found that rs37973 locus mutations were associated with better ICS responses.^31^ Therefore, the relationship between the rs37973 variant and ICS efficacy is inconclusive because of the differences in sample size, ethnicity and efficacy of the evaluation methods.

The Global Initiative for Asthma (GINA) guidelines recommend adjusting asthma treatment according to the degree of daytime and nocturnal symptoms, limitations in physical activity, the need for rescue medication and impairment of lung function.^32^ The main feature of asthma patients is the reversible airflow limitation. The FEV1 and FEV1%pred, which reflect the degree of current airway obstruction, are the most commonly used indicators of lung function to objectively assess asthma status. Clinical studies have suggested that the changes in FEV1 and FEV1%pred in patients compared with their values before treatment can be used to assess whether asthma symptoms are controlled or worsened, thus reflecting the efficacy of drugs for individual patients.^6^, ^33^

In this study, a meta‐analysis was performed to determine the association between the *GLCCI1* (rs37973 G > A) variant and the reduced drug efficacy of ICS in improving lung function in asthmatic patients. The results show that the *GLCCI1* (rs37973 G > A) variant was significantly associated with the FEV1 and FEV1%pred change in the overall analysis. We found that the lung function FEV1 and FEV1%pred changes in patients with the GG phenotype were significantly lower than those in patients with the AA phenotype, and the same results were obtained by the subgroup analysis that followed the duration of treatment. In addition, the FEV1 change in patients with the GG phenotype was significantly lower than that in AG patients. The FEV1%pred change in patients with the AG phenotype was significantly lower than that in patients with the AA phenotype.

The overall analysis of FEV1 changes showed that the GG group had significantly lower changes than the AG groups, whereas there was no significant difference between the GG and AA groups or the AG and AA groups. Izuhara et al. reported that the *GLCCI1* variant is a risk factor for pulmonary function decline in Japanese patients with asthma receiving long‐term ICS treatment.^14^ Another study by Edris et al. confirmed that the minor allele (G) of rs37973 also showed a significantly poorer response to ICS within the Dutch population.^34^ Other previous studies have reported less improvement in the FEV1 before and after treatment in the GG group than in the AA group.^16^, ^22^ This finding was confirmed by this meta‐analysis as well.

Further assessment of the association between the *GLCCI1* (rs37973 G > A) variant and the drug efficacy of ICS was performed using the amount of FEV1%pred change in patients before and after treatment as another outcome indicator. The results of this study showed that the amount of FEV1%pred change in the AA group was significantly better than in the GG group and AG group. Notably, there was no significant difference in the amount of change in the FEV1%pred between patients in the GG and AG groups. Considering that there were only four studies on the amount of change in the FEV1%pred in the GG and AG groups and the AG and AA groups, the small sample size of the studies and the large differences in the included populations may have influenced the statistical power in the combined analysis. Li et al. found that after ICS treatment, the FEV1 and FEV1%pred improved to a greater extent with AA than with AG and GG groups.^24^ The results of this study suggested that patients carrying the G allele may exhibit reduced ICS therapeutic efficacy after ICS administration and that the analysis of *GLCCI1* (rs37973 G > A) in asthma patients will help provide a scientific guidance for the clinical use of ICS.

In the study exploring the rs37973 variant and the efficacy response of ICS, we performed a subgroup analysis of the studies with different follow‐up times to better elucidate the relationship between the rs37973 genotype and the lung function within different treatment times. We found that the FEV1 and FEV1%pred results in the GG and AG groups or the AG and AA groups were similar to the total results, but some differences were detected in the GG and AG groups. The FEV1 and FEV1%pred changes in the GG group were significantly lower than those in the AG groups at 12 weeks. However, the FEV1 at 8 weeks and FEV1%pred at 24 weeks were not significantly different. In both of these subgroups, the age of the study population includes both children and adults. Thus, the rs37973 variant effect might be limited to a more defined population based on a specific age group. However, the inclusion of a range of age groups may have been obscured in the overall meta‐analysis.

However, some potential flaws of this study cannot be ignored when interpreting the results. Although a systematic and comprehensive literature search was conducted, a relatively small number of studies and subjects were included in this study, and a majority of the studies included Asian ethnicity, which may affect the generalization of the findings to other ethnicities. In addition, differences in study ethnicity, population and time of treatment follow‐up may have led to a greater study heterogeneity. Koo et al. found that race influenced the response to glucocorticoids in people with severe asthma compared with white people, and that black patients may have a weakened response to glucocorticoids.^35^ Additionally, age affects the response to systemic corticosteroids in asthma. Phipatanakul et al. found that the use of systemic corticosteroids for severe adult and paediatric asthma significantly reduced disease severity in children without significant changes in adults.^36^ Meanwhile, a recent meta‐analysis also showed that in mild persistent asthma, improved lung function, airway hyperresponsiveness, relief of airway inflammation and symptom control were more significant in children with ICS than in adults.^37^ Therefore, although this paper presents a subgroup analysis based on time of administration, it still cannot eliminate the heterogeneity that exists in the studies, and future work will still require experimentally well‐designed multicentre trials with large sample size to demonstrate the relationship between the rs37973 variant and ICS efficacy.

## 5 CONCLUSIONS

In conclusion, the present meta‐analysis demonstrates that the *GLCCI1* (rs37973 G > A) variant affected the efficacy of ICS in the treatment of asthma. The G allele of *GLCCI1* (rs37973 G > A) might be a risk factor for reduced therapeutic efficacy of ICS. Based on the findings of this study, detecting the genotype of *GLCCI1* rs37973 (G > A) is necessary for patients with asthma who use ICS. In summary, the *GLCCI1* (rs37973 G > A) variant affects the efficacy of ICS in the treatment of asthma, and the results of this study provide a theoretical basis for the use of the rs37973 variant as a predictor of the efficacy of ICS in asthma patients.

## AUTHOR CONTRIBUTIONS

Wen‐Ling Feng conducted the data collection, statistical analysis and wrote the article; Wen Pu, Jing Li and Yuan Yuan collated the data; Ming‐Zhi Yan, Shuang‐Li Yuan, and Yu‐Kun Li developed the search strategy; Ming‐Zhi Yan, Jie‐Ru Wu, and Shao‐Quan Xu conducted the quality assessment; Jun Zhao conceived and designed the study and revised the article. All the authors have read and agreed to the final version of the manuscript.

## CONFLICT OF INTEREST STATEMENT

The authors have no conflicts of interest to declare.

## ETHICS STATEMENT

An ethics statement was not required for this study type as no human or animal subjects or materials were used.

## Data Availability

No additional data available.
